# Should methodological filters for diagnostic test accuracy studies be used in systematic reviews of psychometric instruments? a case study involving screening for postnatal depression

**DOI:** 10.1186/2046-4053-1-9

**Published:** 2012-02-09

**Authors:** Rachel Mann, Simon M Gilbody

**Affiliations:** 1 Department of Health Sciences, University of York, York, YO10 5DD, UK; 2 Hull York Medical School (HYMS), University of York, York, Y010 5DD, UK

**Keywords:** Diagnostic test accuracy, Systematic review, Methodological filters, Literature searching, MEDLINE, Psychometrics, Mental Health

## Abstract

**Background:**

Challenges exist when searching for diagnostic test accuracy (DTA) studies that include the design of DTA search strategies and selection of appropriate filters. This paper compares the performance of three MEDLINE search strategies for psychometric diagnostic test accuracy (DTA) studies in postnatal depression.

**Methods:**

A reference set of six relevant studies was derived from a forward citation search via Web of Knowledge. The performance of the 'target condition and index test' method recommended by the Cochrane DTA Group was compared to two alternative strategies which included methodological filters. Outcome measures were total citations retrieved, sensitivity, precision and associated 95% confidence intervals (95%CI).

**Results:**

The Cochrane recommended strategy and one of the filtered search strategies were equivalent in performance and both retrieved a total of 105 citations, sensitivity was 100% (95% CI 61%, 100%) and precision was 5.2% (2.6%, 11.9%). The second filtered search retrieved a total of 31 citations, sensitivity was 66.6% (30%, 90%) and precision was 12.9% (5.1%, 28.6%). This search missed the DTA study with most relevance to the DTA review.

**Conclusions:**

The Cochrane recommended search strategy, 'target condition and index test', method was pragmatic and sensitive. It was considered the optimum method for retrieval of relevant studies for a psychometric DTA review (in this case for postnatal depression). Potential limitations of using filtered searches during a psychometric mental health DTA review should be considered.

## Background

The advent of systematic reviews has generated challenges to develop optimum methods with which to identify studies from electronic bibliographic databases [1]. There is a great deal of expertise in this matter for systematic reviews of randomised trials [2]. However the design of optimum information retrieval strategies for recent developments such as Diagnostic Test Accuracy (DTA) reviews is not yet resolved; challenges that exist when searching for DTA studies have been acknowledged and include the design of DTA search strategies and selection of appropriate filters [3-5]. DTA studies are important for the assessment of new or existing screening tests; the accuracy of a screening test is assessed by comparing the test to a 'gold standard' to examine if the screening test can accurately classify those with or without the disease, and methodologically rigorous DTA reviews are an important contribution to the overall evidence of a new or existing screening test.

The Cochrane Handbook for Systematic Reviews of Diagnostic Test Accuracy [6] recommends that a search strategy for identification of DTA studies focus primarily on search terms in relation to the 'target condition' (for example, the illness or medical condition) and the 'index test' (for example, the new test to be compared to a gold standard or some other 'reference' test); no specific filter terms such as 'sensitivity' or 'specificity' are recommended. An important contribution to the debate on search filters, by Whiting *et al. *(2010) [7] comprised a substantial review, which sought to clarify the use of search filters by evaluating if an optimum search strategy for retrieval of DTA studies was available. The review found inclusion of filters in DTA searches missed relevant studies; in a head-to head comparison of different strategies the review identified seven systematic reviews, which included 506 primary studies as a 'reference set' to examine the utility and sensitivity of the 'subject search', the search method recommended by the Cochrane Handbook [6] compared to 22 published strategies that used filter terms combined with the 'subject search'. However the reference set only included studies of biochemical laboratory tests such as urinary tract infection, faecal occult blood tests and imaging techniques. When we came to conduct a systematic review of a related but distinct field - psychometrics and the identification of mental disorders - we sought to adapt the approaches of Whiting and colleagues, since their review did not address this issue.

DTA reviews in mental health may present new challenges as the 'index' test will likely be an eponymous psychometric measure, for example the Patient Health Questionnaire-9 [8] or Edinburgh Postnatal Depression Scale [9]. Several studies have specifically examined optimum search strategies for retrieval of mental health studies, however these have focused on the identification of specific study designs, for example intervention studies for mental health or optimum strategies to retrieve papers with content related to a specific mental health condition, for example depression [10-12]. DTA reviews of psychometric measures for depression have been undertaken, however none of the search strategies explicitly described following methodological guidelines [13-16]. In 2007 the National Institute for Health and Clinical Excellence (NICE) issued guidance on the use of questionnaires to detect postnatal depression [17]. In this guidance, they advocate the use of a standardised method to detect postnatal depression and recommend the use of brief case-finding questions:

1) "During the past month, have you often been bothered by feeling down, depressed or hopeless?"

2) "During the past month, have you often been bothered by little interest or pleasure in doing things?"

A third question should be considered if the woman answers "yes" to either of the initial screening questions: "Is this something you feel you need or want help with?"

Recently a DTA review was warranted to evaluate the psychometric properties of these questions and examine the utility of this policy [18]. To our knowledge, the Cochrane recommended search strategy for DTA studies, conducted specifically in the area of psychometrics and mental health has not been evaluated. In this paper we examine whether a useful search strategy is available to identify studies for a DTA review of a brief psychometric measure for postnatal depression.

## Method

A head-to-head comparison of three alternative search strategies undertaken in MEDLINE (1996 to June, week 3, 2011) was compared to a reference set of studies.

### Reference set

A forward citation search using the first publication to conduct a DTA study of the brief psychometric measure as the reference point [19] was performed in ISI Web of Knowledge, which retrieved 350 primary study and review citations. Titles and abstracts were screened for relevant papers. Inclusion criteria consisted of primary studies which examined the brief case-finding questions in a postnatal population, where the accuracy of these questions was compared to a gold standard or 'reference' standard test. A total of six studies which used the case-finding questions for postnatal depression was identified and selected as the reference set [20-25].

### Searches

Each of the three searches contained comparable components in relation to the review question; the searches contained parallel constructs related to the 'target condition' of postnatal depression and parallel constructs related to the 'index test' (the case-finding questions). Search terms related to the index test (the case-finding questions) were constructed by review of titles and abstracts of citations indentified via the forward citation search. Search terms for the target condition (postnatal depression) were constructed from search strings developed for a Health Technology Assessment (HTA) review of postnatal depression [13]. Two of the three searches contained a methodological filter designed to detect DTA studies. The first filtered search was an adapted strategy based on the comprehensive search strategy conducted by University of York, Centre for Reviews and Dissemination (CRD) for the HTA review of methods to identify postnatal depression in primary care [13], hereafter referred to as the 'CRD filter search' (Table 1). The second filtered search used specific filter terms developed by Vincent *et al. *(2003) [26]; their high sensitivity was demonstrated by Richie *et al. *(2007) [27] and these terms are recommended by the Scottish Intercollegiate Guidelines Network (SIGN) [28], hereafter referred to as the 'Vincent filter search' (Table 2). The Cochrane DTA Handbook advises against adding a methodological filter. It uses only two concepts - the 'target condition' and the 'index test'. This was the third search strategy tested, hereafter referred to as the 'Cochrane search' (Table 3). Additional terms for filters, for example use of floating sub-heading 'di.fs' were suggested by an information specialist at the University of York.

**Table 1:** CRD Filter Search conducted in MEDLINE (OVID SP) from 1996 to June Week3, 2011

Searches	Results
1. exp Depression, Postpartum	2427

2. postnatal depress$.mp.	1022

3. post-natal depress$.mp.	54

4. postpartum depress$.mp.	1067

5. post partum depress$.mp.	66

6. (maternal adj3 depress$).mp.	1203

7. (maternal adj3 mental health).mp.	221

8. 1 or 2 or 3 or 4 or 5 or 6 or 7	3872

9. patient health questionnaire-2.mp.	14

10. PHQ-2.mp.	47

11. (2 question or 2 questions).mp.	199

12. (two question or two questions).mp.	1305

13. 2 item$.mp.	338

14. two item$.mp.	648

15. (brief adj3 question$).mp.	1120

16. (case finding adj3 question$).mp.	18

17. (case finding adj3 tool$).mp.	42

18. (screening adj3 question$).mp.	2299

19. (screening adj3 tool$).mp.	7429

20. 9 or 10 or 11 or 12 or 13 or 14 or 15 or 16 or 17 or 18 or 19	12931

21. exp Diagnosis/or diagnosis.mp.	2971479

22. exp Interview, Psychological/or exp Interview/or interview.mp.	58824

23. questionnaire.mp. or exp Questionnaires/	252348

24. mass screening.mp. or exp Mass Screening/	55793

25. 21 or 22 or 23 or 24	3173788

26. di.fs.	905879

27. diagnos$.mp.	911403

28. interview$.mp.	135761

29. question$.mp.	406307

30. screen$.mp.	270644

31. identif$.mp.	1034955

32. predict$.mp.	526679

33. detect$.mp.	817263

34. aware$.mp.	73030

35. assess$.mp.	1106506

36. valid$.mp.	230485

37. 27 or 28 or 29 or 30 or 31 or 32 or 33 or 34 or 35 or 36	3668923

38. 25 or 26 or 37	5051282

39. 8 and 20 and 38	105

[Search 14. di.fs. = search for diagnosis as a floating sub-heading. Annotation mp (multiple posting) = protocol supplementary concept, rare disease supplementary concept, title, original title, abstract, name of substance word, subject heading word, unique identifier]

**Table 2:** Vincent Filter Search conducted in MEDLINE (OVID SP) from 1996 to June Week3, 2011

Searches	Results
1. exp Depression, Postpartum/	2427

2. postnatal depress$.mp.	1022

3. post-natal depress$.mp.	54

4. postpartum depress$.mp.	1067

5. post partum depress$.mp.	66

6. (maternal adj3 depress$).mp.	1203

7. (maternal adj3 mental health).mp.	221

8. 1 or 2 or 3 or 4 or 5 or 6 or 7	3872

9. patient health questionnaire-2.mp.	14

10. PHQ-2.mp.	47

11. (2 question or 2 questions).mp.	199

12. (two question or two questions).mp.	1305

13. 2 item$.mp.	338

14. two item$.mp.	648

15. (brief adj3 question$).mp.	1120

16. (case finding adj3 question$).mp.	18

17. (case finding adj3 tool$).mp.	42

18. (screening adj3 question$).mp.	2299

19. (screening adj3 tool$).mp.	7429

20. 9 or 10 or 11 or 12 or 13 or 14 or 15 or 16 or 17 or 18 or 19	12931

21. exp "Sensitivity and Specificity"/	289302

22. sensitivity.mp.	444219

23. specificity.mp.	440513

24. (predictive adj3 value$).mp.	114342

25. diagnostic error.mp. or exp Diagnostic Errors/	47352

26. ((false adj positiv$) or (false adj negativ$)).mp.	34994

27. (observer adj variation$).mp.	21765

28. (roc adj curve).mp.	20277

29. (likelihood adj3 ratio$).mp.	5304

30. likelihood functions.mp. or exp Likelihood Functions/	11888

31. 21 or 22 or 23 or 24 or 25 or 26 or 27 or 28 or 29 or 30	776221

32. 8 and 20 and 31	31

[Annotation mp (multiple posting) = protocol supplementary concept, rare disease supplementary concept, title, original title, abstract, name of substance word, subject heading word, unique identifier]

**Table 3:** Cochrane Search conducted in MEDLINE (OVID SP) from 1996 to June Week3, 2011

Searches	Results
1. exp Depression, Postpartum/	2427

2. postnatal depress$.mp.	1022

3. post-natal depress$.mp.	54

4. postpartum depress$.mp.	1067

5. post partum depress$.mp.	66

6. (maternal adj3 depress$).mp.	1203

7. (maternal adj3 mental health).mp.	221

8. 1 or 2 or 3 or 4 or 5 or 6 or 7	3872

9. patient health questionnaire-2.mp.	14

10. PHQ-2.mp.	47

11. (2 question or 2 questions).mp.	199

12. (two question or two questions).mp.	1305

13. 2 item$.mp.	338

14. two item$.mp.	648

15. (brief adj3 question$).mp.	1120

16. (case finding adj3 question$).mp.	18

17. (case finding adj3 tool$).mp.	42

18. (screening adj3 question$).mp.	2299

19. (screening adj3 tool$).mp.	7429

20. 9 or 10 or 11 or 12 or 13 or 14 or 15 or 16 or 17 or 18 or 19	12931

21. 8 and 20	105

[Annotation mp (multiple posting) = protocol supplementary concept, rare disease supplementary concept, title, original title, abstract, name of substance word, subject heading word, unique identifier]

### Analysis

The performance of the three searches was compared to the reference set as described by Whiting *et al. *[7], in terms of completeness of retrieval; the sensitivity of each search (number of relevant studies/reference set × 100) and missed studies (reference set - number of relevant studies identified) was calculated. Efficiency of the assessment process for the researcher was assessed using precision (number of relevant studies/total number of studies identified in search × 100). Associated 95% confidence intervals were calculated using the Vassar online statistics calculator for proportions [29].

## Results

**Figure 1:**
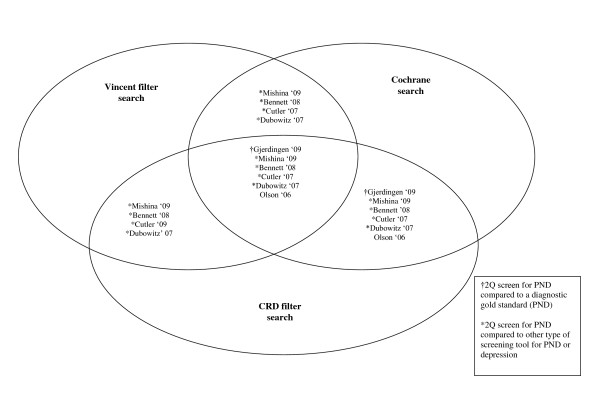
**Head to head comparison of three alternative search strategies conducted in Medline 1996 to June, Week 3 2011**.

Table 4 presents the results of the head-to-head comparison of the three strategies. The Cochrane search and CRD filter search each retrieved a total of 105 citations; precision was low (5.2%) due to the number of irrelevant studies identified, however both searches were 100% sensitive and retrieved all reference set studies. The Vincent filter search retrieved a total of 31 citations; although precision was slightly higher due to the smaller number of studies retrieved, the search had poor sensitivity. Of the six papers identified as the reference set, only one paper examined the DTA of the questions compared to a 'gold standard' diagnostic criteria [20], therefore only this study would be eligible for inclusion in a DTA review of the questions. Whilst the Cochrane search and CRD filter search identified this study [20] the Vincent filter search failed to do so. A Venn diagram (Figure 1) shows the commonality and discrepancy between the three searches in MEDLINE.

**Table 4:** Summary table of the performance of the three search strategies

Search Strategy	Total number of records retrieved	Number of reference set studies missed	Sensitivity (%) (95% CI)*	Precision (%) (95% CI)*
Cochrane search	105	0	100 (61, 100)	5.7 (2.6, 11.9)

CRD filter search	105	0	100 (61, 100)	5.7 (2.6, 11.9)

Vincent filter search	31	2	66.6 (30, 90)	12.9 (5.1, 28.6)

*[95% confidence interval]

## Discussion

This small exploratory case study sought to identify a useful search strategy for identification of DTA studies of a brief psychometric measure recommended for identification of postnatal depression. Relatively few DTA reviews have been conducted in mental health, and those that have addressed this area do not describe a formal method to construct their search strategy. Studies that have examined the sensitivity and accuracy of various search strategies to inform search techniques for DTA reviews, including current published Cochrane DTA Reviews [6] tend to focus on DTA of physical and laboratory tests. In a review of 12 search filters for retrieval of DTA studies, Leeflang *et al. *[4] included 27 diagnostic systematic reviews, of which one review examined the DTA of two measures to identify alcohol problems in primary care, however, to our knowledge, no paper has specifically examined if a useful search strategy to conduct a DTA review of a psychometric measure for a mental health disorder was available.

Three strategies with alternative methods and search terms were compared to a reference set of records. The Cochrane search and CRD filter search identified all studies in the reference set. Despite the complex use of a variety of filter terms within the CRD filter search, the total number of records retrieved and the completeness of retrieval for relevant studies from the Cochrane search (no methodological filter included) was identical. The construction of the strategies in relation to the use of filters was different, yet the Cochrane search did not suffer loss of any relevant studies. Therefore it had the advantage of providing a less complex yet more pragmatic search strategy, which could be applied to a wide range of electronic databases with the potential for low risk of missing key citations. A potential consequence of not adding a methodological filter when searches for studies are conducted in a large evidence base is the potential loss of precision. Low precision was observed with the Cochrane search. Searches where researchers have many citations to screen in order to identify relevant studies may not be viewed as the most efficient strategy. There is a potential trade off between high sensitivity and low precision that needs consideration with this approach. This may point to further work to balance the need for precision, the pragmatism of less complex searches (as recommended by the Cochrane Group) and the need to refine filter terms related to psychometric tests. The Vincent filter search included specific filter terms to identify DTA studies, so precision was slightly higher than that observed with the Cochrane search. At the same time, sensitivity was lower in comparison with the Cochrane search. As such, the Vincent filter search failed to retrieve the most important study to the review, a DTA study which used gold standard diagnostic criteria [20]. A possible explanation for this is mental health DTA studies are likely to suffer from indexing problems under the filter terms in MEDLINE in the same way as studies of biochemical, laboratory tests or imaging tests do. Indexing problems of filter terms associated with the Vincent filter search might explain the low number of retrievals using the specific DTA filter terms. Additionally, psychometric tests are often associated with terms related to reliability and validity rather than diagnostic accuracy terms. Richie et al (2007) [27] has acknowledged the difficulty of transcription, indexing and lack of transparency in reporting search filters in papers and electronic sources used to conduct DTA searches. Use of the Standards for the Reporting of Diagnostic Accuracy Studies (STARD) [30] guidelines to ensure accuracy, transparency and completeness of reporting DTA studies may assist indexing and identification of psychometric DTA studies in electronic databases, as STARD recommends use of diagnostic terminology for example, sensitivity and specificity and so on for all studies reporting diagnostic accuracy. In addition, a recent and very positive development by EMBASE is the addition of a diagnostic test accuracy indexing term in EMTREE, which should assist researchers searching for DTA studies in this particular database [31].

This was a small exploratory search with limitations, for example the reference set may not be considered a 'gold standard' comparator as it was derived from a forward citation search. However, there are very few DTA reviews of psychometric measures for postnatal depression [18,13], so there was a relatively small chance of missing relevant studies in this specific area. Evaluation of the Cochrane search strategy for DTA reviews would benefit from further consideration in other mental health disorders and alternative electronic databases as it is difficult to generalise the results of this small study as a method of retrieval of psychometric DTA studies for all mental health conditions.

## Conclusions

The findings from this exploratory study reflect the conclusions of Whiting *et al. *[7] and in our case, the 'target condition and index test' Cochrane search provided an effective and pragmatic strategy when constructing a systematic search strategy for retrieval of DTA studies of a brief psychometric measure for postnatal depression in electronic databases. In addition, use of the Cochrane search provided a means to report the conduct of the DTA review search strategy with transparency, according to published guidelines. It is therefore reasonable to conclude that researchers may find it preferable to consider use of the 'target condition and index test' method recommended within the Cochrane Handbook for Systematic Reviews of Diagnostic Test Accuracy [6] rather than filtered searches for a DTA search strategy of a psychometric measure for mental health disorders such as postnatal depression.

## List of abbreviations

95% CI: 95% confidence interval; CRD: Centre for Reviews and Dissemination; DTA: Diagnostic Test Accuracy; HTA: Health Technology Assessment; NICE: National Institute for Health & Clinical Excellence; SIGN: Scottish Intercollegiate Guidelines Network.

## Competing interests

The authors declare that they have no competing interests.

## Authors' contributions

RM designed the concept and undertook the searches and drafted the final manuscript. SG commented on, and helped draft the final manuscript. All authors read and gave approval to the final manuscript.

## Author's Information

The decision to choose an appropriate search strategy was faced by the researcher (RM) when they came to conduct a DTA review of a psychometric measure in postnatal depression. As definitive guidelines for conduct of diagnostic reviews were published last year in the first Cochrane Handbook for Systematic Reviews of Diagnostic Test Accuracy (2010), the researcher was interested to know if the Cochrane recommended search for diagnostic reviews was relevant to a psychometric diagnostic review in mental health - the complexity/pragmatic aspects faced when using multiple, unfamiliar search filters compared to a brief, relatively simple method was of interest. Overall, the ability to justify the search strategy used in the review was deemed important in terms of reporting transparency as other psychometric diagnostic reviews in mental health have not specified a specific strategy method or cited specific guidance for their strategy choice.
